# Newcomb-Benford number law and ecological processes

**DOI:** 10.1371/journal.pone.0310205

**Published:** 2025-03-28

**Authors:** Robert D. Davic

**Affiliations:** State of Ohio Environmental Protection Agency, Columbus, Ohio, United States of America; Universidad de Valladolid, SPAIN

## Abstract

The Newcomb-Benford number law has found applications in the natural and social sciences for decades, with limited ecological attention. The aim of this communication is to highlight a statistical correspondence between the first significant digit frequencies of the Benford probability distribution and ecological systems in a balanced state of dynamic equilibrium. Analytical methods, including multidimensional Euclidean distance, Cohen-W effect size, Kossovsky sum of squared deviations (SSD), and Pearson residuals are presented to facilitate the identification of this canonical representation across multiple levels of ecological organization and scale. Case studies reveal novel applications of the Benford distribution for detecting impending state transitions in marginally stable systems, as well as temporal and spatial divergence of ecological information through the measurement of Kullback-Leibler relative entropy. Widespread documentation of the leading digit phenomenon is expected as ecologists revisit empirical datasets and formalize sampling protocols for its detection. The conversion of randomly collected sets of arithmetic data into logarithmic probabilities of first significant digits presents unique opportunities to advance our understanding of ecological processes related with stability, complexity, and maturity.

## Introduction

Ecological systems are organized by a combination of negative and positive feedback mechanisms involving organisms and their abiotic environment [1]. The blend of random and non-random (natural selection) interactions generates interest to identify self-organizing patterns that unify ecological processes across multiple levels of organization and scale [2]. Pattern derived from empirical data has been linked to mathematical distributions with ‘law-like’ characteristics [3–6], an area not extensively explored in ecology [7].

**Fig 1:**
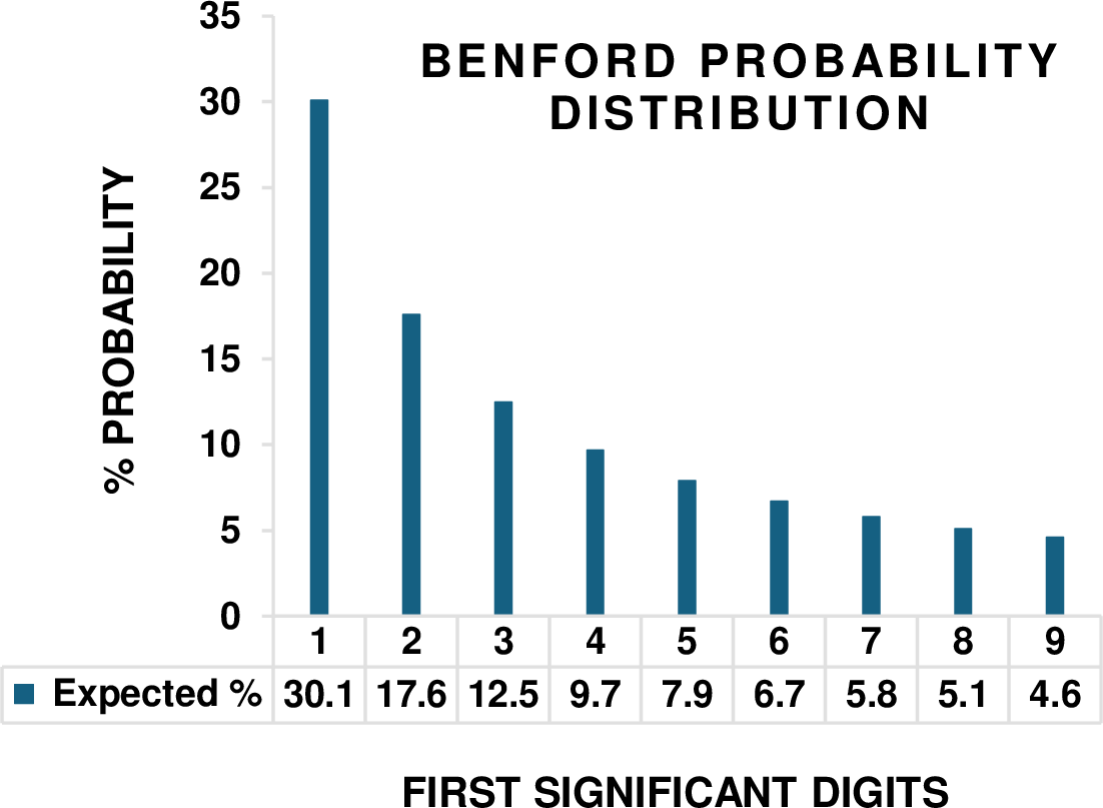
The Benford probability distribution of first significant digits derived from a set of naturally occurring empirical data. Probability *(d)* =  log_10_ [1 + (1/*d*)], where (*d* ∈  {1...9}).

The aim of this communication is to highlight correspondence between the well-established Newcomb-Benford number law (Newcomb, 1881 [8], Benford, 1938 [9], Pinkham, 1961 [10]), and ecological processes related with stability, complexity, and maturity. The Benford probability distribution (aka, Benford’s law) is an empirical observation that a first significant digit (FSD) transformation of a naturally occurring dataset (i.e., with minimal human-induced alteration) exhibits a discretely skewed pattern of logarithmic probabilities for certain measurement variables (Fig 1, Table 1). This leading digit phenomenon has been applied in the natural and social sciences for decades [11–25]. Both its emergence from a set of empirical data, and distortion once formed, can signal abnormal system behavior. Examples of the former include the appearance of the Benford distribution in response to seismic waves at the onset of earthquakes [11], and arrhythmia related to cardiac activity [12]. Conversely, in economics, auditors identify discrepancies of numeric values on spreadsheets from expected Benford’s law first digit frequencies to detect fraud in data tabulation [13]. Normal pattern of electrical brain activity conforms with the Benford distribution until altered by the administration of sevoflurane anaesthetics [14].

**Table 1:** First significant digit relative frequencies for the Benford distribution.

First Digit	Relative Frequency	Fractional
1	0.30103	log_10_ (2/1)
2	0.17609	log_10_ (3/2)
3	0.12494	log_10_ (4/3)
4	0.09691	log_10_ (5/4)
5	0.07918	log_10_ (6/5)
6	0.06695	log_10_ (7/6)
7	0.05799	log_10_ (8/7)
8	0.05115	log_10_ (9/8)
9	0.04576	log_10_ (10/9)

Unexplored potential for ecological application arises from reports associating the Benford distribution with attributes of steady state dynamic equilibrium for naturally occurring systems [13,14,16–18], defined by Thoms et al. [26] as balanced long-term fluctuations about short-term constantly changing conditions. The recurring theme is that fluctuations in system processes essential for maintaining steady state conditions can be quantified by analyzing the statistical distance between two probability distributions: one derived from a set of empirical measurements converted into first digit probabilities, and the other following the internally consistent pattern of logarithmic probabilities described by the Benford distribution (Table 1). The correspondence is identified with thermodynamic theory [27,28] and information entropy [16,17], subjects with broad ecological applications [29–33]. These findings suggest a hypothesis that, within the context of ecological systems open to the flux of matter and energy, a state transition either toward or away from steady state behavior can be quantified by analyzing the pattern of disorder (entropy) between observed first digit probabilities and Benford’s law expected probabilities in response to environmental changes. Prigogine and Nicolis [34] introduced the concept of ‘biological dissipative structures’ to describe systems that maintain their steady state structure by minimal entropy production, which also has been applied to evolutionary transitions [35]. This description aligns with the Ulanowicz’s ascendency view of ecological stability [36], defined as the tendency of ecosystem processes “to remain within an arbitrarily designated nominal range of behaviors” in response to changing environmental conditions.

Anecdotal evidence that ecological systems in steady state dynamic equilibrium adhere to the first digit probabilities described by the Benford distribution comes from genomic reading-frame sequences (ORFs) that exhibit persistent leading digit gene patterns over evolutionary time scales that distinguish taxonomic categories above the species level [17], consistent daily mean pollen counts from multiple atmospheric sample locations [18], and seasonally predictable communities of lake algae [37]. Naturally assigned taxonomic categories of angiosperms (species and family level) conform with the Benford distribution while those artificially assigned (genus) do not [38]. Pröger et al. [39] applied a Benford’s law first digit analysis to telemetry data for wild red deer freely moving in natural habitats. Ninety-one percent of 1132 weekly sets of data conformed with the Benford distribution. Disturbance from human activity was identified as a possible causal factor for non-conformity. Naturally occurring bird species datasets, when measurements are log-normally distributed and rare species are included in the tally, adhere to the Benford’s law first digit pattern [40], as well as consistent thresholds of occurrence sites for modelling species distributions [41].

After review of the Newcomb-Benford number law, empirical data taken from the ecological literature are presented to test the proposition that measurement variables for systems reported to be in a balanced state of dynamic equilibrium, or in theory progressing toward steady state behaviour, can be well approximated by the internally consistent pattern of first digit probabilities predicted by the Benford probability distribution (Fig 1, Table 1). The proposition was tested analytically using sets of measurement variables for ecological systems spanning multiple levels of organization and scale: 1) annual biomass of below ground functional groups during developmental stages of ecological succession [42]; 2) cell counts for lotic diatom species colonizing artificial habitats (glass slides) and progressing in theory toward a steady state of species richness [43]; 3) long-term persistence in abundance of multiple species of woodland salamanders over a wide geographic area [44]; and 4) the relative weight (biomass) of stream fish species from five geographically isolated communities with consistent and exceptional biotic integrity [45].

## Methods

### Background

The Newcomb-Benford number law [8–10], also known as the first digit law and Benford’s law (herein BL), is a mathematical oddity in the probability of first significant (non-zero) digits observed from certain sets of empirical data (Fig 1). The discretely skewed Benford probability distribution emerges when a set of random measurements for a system variable, such as biomass of herbivorous insect species in a grassland habitat (0.061, 0.14, 9.21, 20.2, 23.0, 723.0, 3345.0…), are transformed into a set of first significant digits (6, 1, 9, 2, 2, 7, 3…). The expected probability P(*d*) of leading digits conforms to the logarithmic equation:

$$P\left(d\right)=\log_{10}\left[1+\left(1/d\right)\right],where\left(d\in \left\{1\ldots 9\right\}\right)$$ (1)

where the digit #1 appears about 30% of the time, digit #9 near 5%, with a log_10_ sequence of the nine first digit probabilities (Table 1).

First presented by Newcomb in 1881 [8], the leading digit phenomenon was rediscovered in 1938 by Benford [9] and empirically verified from 20,000 + measurements taken on 20 sets of naturally occurring data compiled across multiple fields of study. While the skewed first digit Benford distribution (Fig 1) is more widely applied in the literature [11–25], the initial mathematical representation of Newcomb [8] identified a uniform distribution of leading digits from analysis of the first fractional component of the mantissa (0.1 to 0.9) after a log_10_ transformation of a given measurement. The Benford distribution is uniquely both scale [10] and base [46] invariant. Increased statistical conformity is expected when data from multiple measurement variables are aggregated, a phenomenon first reported by Benford [9] by combining data from twenty independent variables. Additional information from the analysis of second and third significant digits can improve conformity for some dataset applications but requires significantly larger sample size [13]. The most common application is in economics, where deviation from an expected BL pattern of first digits is used to detect errors and biases in data collection and reporting [13,47]. Mathematical reviews include Berger and Hill [48], Miller [49], Fang and Chen [50]. A real-time online bibliography of Berger, Hill, and Rogers [51] provides 2200+ articles and books with theoretical and applied examples: https://www.benfordonline.net; date accessed 09/24/2024.

While the leading digit phenomenon is commonly associated with exponentially growing and decaying processes [48–50], many empirical datasets do not conform to the Benford probability distribution [52]. An underlying explanation can be attributed to constraints related to the assessment of data, which serve as preconditions for conformity. Constraints play an important role when analyzing the statistical relationship between observed and expected probability distributions, as they help to define the range of possible outcomes that lead to conformity (Ashby, p. 127–134, [53]). Zwick [54] surmises that constraints on possible variety add practical meaning to information. An increase in the number and extent of *constraint deviations* lessens the practical likelihood the Benford distribution will emerge from a set of empirical data. Assessment preconditions this author deems pertinent for ecological applications include: 1) The collection of data follows methods of random sampling where each of the nine first digit categories has an equivalent chance of occurrence for each measurement taken, with sample locations selected without prior bias to exclude habitat heterogeneity. The mathematical theorem of Hill [52] highlights these criteria: “*if distributions are selected at random (in any unbiased way) and random samples are taken from each of the distributions, then the significant digit frequencies of the combined samples converge to Benford’s distribution though the individual distributions selected may not closely follow the law”*; 2) The dataset spans at minimum one order of magnitude with sufficient sample size to populate all nine first digit categories [48]. Six orders of magnitude were suggested by Fewster [55] to increase the likelihood for emergence of the BL leading digit pattern, but as few as two reported here; 3) The dataset derives from natural processes with minimal direct human disturbance, such as the imposition of arbitrary thresholds on data analyzed [38], or alteration of environmental conditions that can distort an observed first digit pattern from naturally occurring expectations [39–41]; 4) The tabulation of data is free from intentional human manipulation [13,47]; 5) Tabulated data are generated by non-additive mathematical operations such as multiplication, division, or numbers raised to integer powers [56].

### Statistical analysis

The American Statistical Association (ASA) cautions against relying on statistical *p*-values as a stand-alone indicator of evidence when drawing conclusions from analysis of empirical data [57]. To enhance the reliability of conclusions reached from the analysis of reviewed case reports, a weight-of-evidence approach was adopted by applying multiple analytical methods. Data tables for each case study were calculated from an Excel spreadsheet using the formulas given for each analytical test method.

#### Analytical method #1.

For statistical inference, the following null hypothesis (H_O_) assumption was tested: the observed macroscopic first significant digit probability sequence is well approximated by the sequence expected by the Newcomb-Benford number law (Eq. 1, Table 1), with alternative hypothesis (H_A_): the observed first digit sequence is not well approximated. The (H_O_) was analyzed using a nine-dimensional Euclidean distance formula, recommended for BL application by Cho and Gaines [58], as modified by Morrow [59] to allow for statistical inference:

$$MorrowEuclidean\text{ Distance d}{*}_{n}=(\sqrt{n})\sqrt{\overset{9}{\underset{d=1}{\sum }}\left[OBS\left(d\right)–EXP\left(d\right)\right]²}$$ (2)

where *d* =  first significant digits (1:9), n =  sample size of observations, OBS =  observed probability a random measurement *x* has a first digit =  *d* for total sample size n, and EXP =  BL expected first digit probabilities (Table 1). An asymptotic rejection range (alpha 0.10, d * _n_ =  1.22; alpha 0.05, d * _n_ =  1.33; alpha 0.01, d * _n_ =  1.57) is presented by Morrow using Monte Carlo simulation for datasets with sample size from 80 to 500. A rejection value d * _n_ ≥  1.33 was adopted to test the (H_O_). A signal of impending state transition in alignment with conformity to the Benford distribution was established as (1.22 <  d * _n_ <  1.33).

#### Analytical method #2.

Kossovsky [60] advocates use of sum of squared deviations (SSD) to quantify the descriptive distance between measured data with BL first digit expectations, without reference to sample size and use of *p*-values. The SSD is calculated as:

$$KossovskySSD=\overset{9}{\underset{d=1}{\sum }}{\left[\left(OBS\left(d\right)-EXP(d\right)\right]}^{2}x{\text{ 10}}^{4}$$ (3)

with symbols as given for (Eq. 2). Confidence intervals (95%) were calculated using the Excel command [=Confidence.T (0.05, (STDEV.S), 9)], with standard deviation calculated from 1000 bootstrap resamples [Statkey, v.3.04, 2024; https://www.lock5stat.com/Statkey]. Kossovsky proposed SSD ≥  100 as a descriptive measure a set of quantitative data does not conform with the Benford distribution, while SSD ≤  25 suggests moderate to strong conformity. A signal of impending state transition was established as (75 < SSD <  100; Kossovsky personal communication).

#### Analytical method #3.

Cohen [61] recommends calculation of effect size to estimate the practical importance of deviation between two probability phenomena to complement interpretation of results from statistical inference. A greater distance between observed and expected Benford distributions would be associated with a larger Cohen-W effect size, with ‘effects’ related to the practical number and magnitude of constraint deviations associated with the BL assessment process (see Methods section). One measure of effect size that is independent of sample size and complements information from Euclidean distance is the Cohen-W:

$$Cohen-W=\sqrt{\overset{9}{\underset{d=1}{\sum }}[{\left(OBS\left(d\right)–EXP\left(d\right)\right)}^{2}/EXP\left(d\right)]}$$ (4)

with symbols as given in (Eq. 2). Confidence intervals (95%) were calculated using the Excel command [=Confidence.T (0.05, (STDEV.S), 9)], with standard deviation calculated from 1000 bootstrap resamples [Statkey, v.3.04, 2024; https://www.lock5stat.com/Statkey]. Cohen offers descriptive thresholds to identify weak, moderate, and strong effect sizes respectively (W =  0.1, 0.3, 0.5). A Cohen-W ≥  0.5 provides strong descriptive evidence of non-conformity with the Benford distribution. A signal of impending state transition was established as (0.3 <  W <  0.5).

#### Analytical method #4.

The microscopic variability of individual first digit relative frequencies was statistically estimated from the Pearson residual (PR) formula presented by Sharpe [62]:

$$PR=\left(\left(OBScount\right)-\left(EXP\text{ count}\right)\right)/\sqrt{EXPcount}$$ (5)

where OBS =  measured first digit count frequency, and EXP =  predicted first digit frequency based on total sample size n (i.e., from Table 1, the EXP count for digit #1 = 0.30103 * n). The presence of two or more Pearson residual values ≥  1.96 (Z-Test, *p* =  0.05) was adopted as evidence of statistical microscopic non-conformity with the Benford distribution, with one Pearson residual ≥  1.96 a signal of impending state transition.

#### Analytical method #5.

The Kullback-Leibler divergence (KL_D_), also known as relative entropy, is a measure from information theory [63] used to quantify similarity (dissimilarity) of ecological information between two probability distributions [64, 65]. Although KL_D_ does not directly estimate the absolute quantity of Shannon-Wiener information within a system, it can be applied to quantify the transition of relative entropy (the measure of information loss or disorder) in response to fluctuating environmental conditions [64], or between two spatial patterns [65]. In the context of a BL leading digit analysis, the higher the KL_D_ between observed and expected first digit probability distributions the more pronounced the dissimilarity in ecological information.

To examine correspondence between analytical measures of statistical and descriptive distance with results obtained from calculation of Kullback-Leibler divergence, the discrete KL_D_ formula presented by Huckeba et al. was adopted [65], which this author has symbolically modified for BL application:

$$KL\text{ Divergence }\left(obs∥\exp\right)=\overset{9}{\underset{d=1}{\sum }}OBS\left(d\right)*\left[\log_{10}\frac{OBS\left(d\right)}{EXP\left(d\right)}\right]$$ (6)

where OBS (*d*) and EXP (*d*) are as given in (Eq. 2), and (obs||exp) measures the divergence of relative entropy (directional loss of information) when comparing the first digit probabilities of empirical data (OBS) to the expected reference approximation of the Benford distribution (EXP). A KL_D_ of zero signifies complete similarity of information entropy between distributions. The Percentage Difference formula: (PD =  | a - b | / [(a +  b)/ 2] * 100) estimates the magnitude of divergence between two sample events (a and b), with PD <  10% adopted as a nominal level of KL_D_ dissimilarity.

## Results

The presentation of Results is divided into two sections. A description of analytical test results for each of the reviewed case reports is followed by a comparison of the case report findings.

### Case Report #1. Food-web stability during ecological succession

Neutel et al. [42] sampled two below ground food-webs representing four developmental stages of succession at the Schiermonnikoog [SCH] island national park and Hulschorsterzand [HUL] mainland nature reserve, Netherlands. Food-web stability was quantified from dynamic models related to three omnivorous feedback loops. Non-random predator-prey interactions within these feedback loops were identified by Neutel et al. to play an important role in maintaining food-web stability at both sample locations, as productivity and functional group complexity increased during succession.

The focus of this case report was to determine if the food-web stability for the two natural areas reported by Neutel et al. can be well approximated by the first digit pattern described by the Benford probability distribution. Mean annual biomass for fifteen functional groups (gms C ha^-1^ cm depth^-1^, their Suppl. Table-1), collected from four developmental stages of succession, meet data assessment constraints necessary to test conformity with the Benford distribution (see Methods section). Soil samples were randomly collected three times in upper soil layers with four replications per development stage to establish annual mean biomass per functional group. The dataset spans six orders of magnitude at both sample locations. Sufficient sample size (n ≥  80) to apply Morrow Euclidean distance was available for statistical analysis when biomass data are combined for the SCH and HUL sample locations and segregated into early (1, 2) and late (3, 4) developmental stages of succession. Minimal anthropogenic disturbance is expected for the two natural areas.

**Fig 2:**
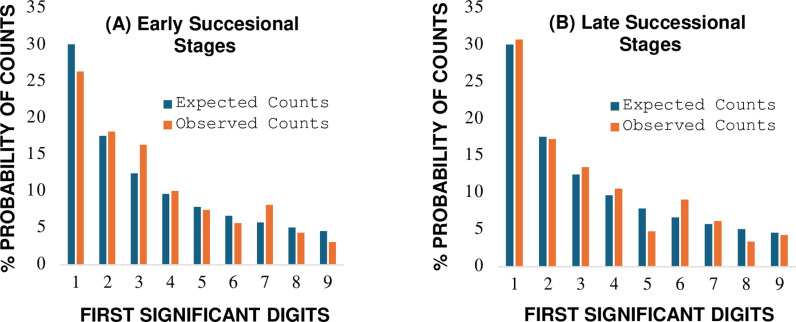
Comparison between observed first digit counts and Benford’s law expected counts for stable below ground food-webs during (A) early and (B) late developmental stages of succession. Graphs of data from Table 2.

The weight of evidence from analytical tests (Table 2, Fig 2) supports the tested proposition that mean annual biomass of stable below ground function groups, at the combined SCH and HUL natural areas, is well approximated by the Benford probability distribution. No analytical signals of impending transition away from BL reference expectations are indicated. The consistency in the pattern of first digit frequencies between early and late stages of succession (Fig 2) aligns with findings of Neutel et al. that below ground food-webs at the two natural areas have maintained a balanced state of dynamic equilibrium over time.

**Table 2:** Observed first digit counts of mean annual biomass for fifteen functional groups *vs* Benford’s law expected counts. Data from combined SCH and HUL sample locations. Raw data from Neutel et al., [42].

First Digit	1	2	3	4	5	6	7	8	9
Early succession (development stages 1 & 2)									
OBS Count	42	29	26	16	12	9	13	7	5
EXP Count	47.9	28.0	19.9	15.4	12.6	10.6	9.2	8.1	7.3
Pearson Residual	-0.85	+0.19	+1.38	+0.15	-0.17	-0.50	+1.24	-0.40	-0.84
% OBS Probability	26.4	18.2	16.4	10.1	7.5	5.7	8.2	4.4	3.1
% EXP Probability	30.1	17.6	12.5	9.7	7.9	6.7	5.8	5.1	4.6
FSD sample size n = 159. Max Pearson residual = 1.38; Euclidean distance d * _n_ = 0.782 (p > 0.10); Cohen-W = 0.184, 95% CI [0.181, 0.186]; Kossovsky SSD = 38.4, 95% CI [37.9, 38.9]; Kullback-Leibler divergence (0.0071 Hartley units).									
First Digit	1	2	3	4	5	6	7	8	9
**Late succession (development stages 3 & 4)**									
OBS Count	64	36	28	22	10	19	13	7	9
EXP Count	62.6	36.6	26.0	20.2	16.5	13.9	12.1	10.6	9.5
Pearson Residual	+0.18	-0.10	+0.39	+0.41	-1.59	+1.36	+0.27	-1.12	-0.17
% OBS Probability	30.8	17.3	13.5	10.6	4.8	9.1	6.2	3.4	4.3
% EXP Probability	30.1	17.6	12.5	9.7	7.9	6.7	5.8	5.1	4.6
FSD sample size n = 208. Max Pearson residual = 1.59; Euclidean distance d * _n_ = 0.664 (p > 0.10); Cohen-W = 0.171, 95% CI [0.168, 0.175]; Kossovsky SSD = 21.2, 95% CI [18.7, 23.7]; Kullback-Leibler divergence (0.0068 Hartley units).									

Results from analysis of Kullback-Leibler relative entropy show a nominal percentage difference (PD =  4.32%) in the divergence of ecological information related to production of food-web biomass between early and late developmental stages of succession (KL_D_: early =  0.0071, late =  0.0068 Hartley units, Table 2). The KL_D_ values are associated with full analytical conformity to the BL reference approximation. Neutel et al. [42] demonstrated that non-random stabilizing effects on food web interactions from omnivore feedback loops offer one causal mechanism to help explain the similarity in the divergence of Kullback-Leibler relative entropy between early and late developmental stages of succession.

Altena et al. [66] revisited the study of Neutel at al. [42] and recommended use of ‘biomass flux rate’ as an alternative measurement variable to omnivore feedback loops to assess food web stability at the individual SCH and HUL locations. The study was motivated in part from reports in the literature that species interactions in ecosystems are not evenly distributed in space and time, but instead follow power law patterns of probability. The case study results presented here align with this observation. The flux of mean annual biomass for multiple functional groups over successional time conforms with the skewed Benford probability distribution (Fig 2), a discrete distribution strongly correlated with a power law function (R^2^ =  0.998, n =  9, Table 1 data). Future research utilizing a BL leading digit analysis of biomass flux rate is recommended to investigate the suggestion of Altena et al. that food web stability has increased over successional time at the individual SCH and HUL natural areas, a trend implied from the non-overlapping 95% confidence interval estimates of Cohen-W and Kossovsky SSD for combined sample locations (Table 2).

### Case Report #2. Dynamic equilibrium of diatom colonization

MacArthur and Wilson [43], their Table 6, provide diatom counts (the number of cells per diatom species) for a colonization experiment conducted by Ruth Patrick at Roxborough Spring, Pennsylvania. Over two weeks, diatoms colonized glass slides representing two sizes of ‘islands’ (12 & 25 mm^2^). Replicate samples include experiments 1 & 3 (small islands) and 2 & 4 (large islands). By experiment end, the combined data showed a diverse community of 54 diatom species and >600,000 cell counts. MacArthur and Wilson cited the Patrick data to support their equilibrium theory of island biogeography [43], which predicts the colonization process on the different sized islands was progressing toward a steady state of diatom species richness in response to selective pressures related to rates of species immigration and extinction.

The focus of this case report was to determine if the diatom communities colonizing the two differently sized islands are well approximated by the Benford probability distribution. The Patrick dataset meets data assessment constraints required to test correspondence with BL expectations (see Methods section). Diatom counts span six orders of magnitude with sufficient sample size (n ≥  80) to test conformity using Morrow Euclidean distance, when results from the one-and two-week replicated experiments for same sized islands are combined. While details of sampling methods used by Patrick are not presented it is assumed diatom colonization was quantified, using a floating Diatometer chamber as described elsewhere by Patrick [67], from the average cell counts per island estimated from sub-samples of organisms recovered from cleared glass slides. Patrick identified Roxborough Spring as perennial flowing and free from human disturbance [67].

**Fig 3:**
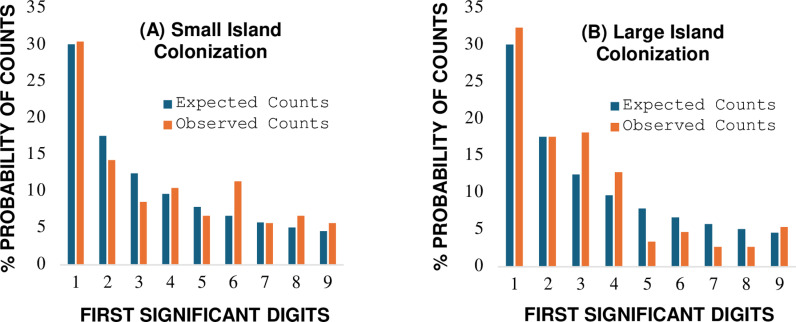
Comparison between observed first digit counts and Benford’s law expected counts for diatom species colonizing glass slide islands at (A) small and (B) large spatial scales. **Graphs of data from Table 3**.

Analytical test results show differential outcomes based on space available for diatom colonization (Table 3, Fig 3). The weight of evidence for *small* islands supports an interpretation the colonization process for diatom communities is well approximated by the Benford probability distribution. No analytical signals of impending state transition away from BL reference expectations are indicated. The pattern of observed *vs* BL expected first digit probabilities associated with diatom colonization (Fig 3) is similar to the successional trend observed for stable below ground food-webs (case report #1, Fig 2). These findings suggest an interpretation the diatom communities on the smaller islands had attained a balanced state of dynamic equilibrium after the two-week colonization process. Independent support comes from research by Patrick [67] at Roxborough Spring where the species richness of diatom taxa after a two-week span of colonization was similar to values recorded from experiments extended to eight weeks.

**Table 3:** Observed first digit diatom community cell counts *vs* Benford’s law expected counts for colonization experiments. Data from MacArthur and Wilson [43], their Table 6.

First Digit		1	2	3	4	5	6	7	8	9
Experiments 1 & 3 (12 mm^2^ slides, ‘small islands’)										
OBS Count		32	15	9	11	7	12	6	7	6
EXP Count		31.6	18.5	13.1	10.2	8.3	7.0	6.1	5.4	4.8
Pearson Residual		−0.07	−0.81	−1.14	+0.26	−0.46	+1.87	−0.04	+0.70	+0.55
% OBS Probability		30.5	14.3	8.6	10.5	6.7	11.4	5.7	6.7	5.7
% EXP Probability		30.1	17.6	12.5	9.7	7.9	6.7	5.8	5.1	4.6
FSD sample size n = 105. Max Pearson residual = 1.87; Euclidean distance d * _n_ = 0.759 (p > 0.01); Cohen-W = 0.250, 95% CI [0.242, 0.257]; Kossovsky SSD = 54.9, 95% CI [48.1, 61.7]; Kullback-Leibler divergence (0.0125 Hartley units).										
First Digit	1	2	3	4	5	6	7	8	9	
**Experiments 2 & 4 (25 mm** ^2^ ** slides, ‘large islands’)**										
OBS Count	48	26	27	19	5	7	4	4	8	
EXP Count	44.6	26.1	18.5	14.3	11.7	9.9	8.6	7.6	6.8	
Pearson Residual	+0.52	−0.01	**−1.98**	+1.23	**−1.96**	−0.92	−1.56	−1.30	+0.47	
% OBS Probability	32.4	17.6	18.2	12.8	3.4	4.7	2.7	2.7	5.4	
% EXP Probability	30.1	17.6	12.5	9.7	7.9	6.7	5.8	5.1	4.6	
FSD sample size n = 148. Max Pearson residual = (**1.96**, **1.98)**; Euclidean distance d * _n_ = 1.147 (p > 0.10); Cohen-W = **0.316,** 95% CI [0.308, 0.323]; Kossovsky SSD = **88.9,** 95% CI [79.7, 98.2]. Kullback-Leibler divergence (0.0238 Hartley units). Bold values for Cohen-W & SSD suggest impending state transition. Pearson residuals indicate non-conformity with the Benford distribution at *p* < 0.05.										

In contrast to results for smaller islands, analytical results for *larger* islands showed marginal macroscopic conformity to the Benford distribution with higher levels of first digit volatility for Cohen-W effect size (W =  0.316) and Kossovsky sum of square deviations (SSD =  88.9), values within adopted ranges of state transition either toward or away from BL reference thresholds (Table 3, Fig 3). Volatility of Pearson residuals showed microscopic non-conformity to the Benford distribution (Table 3). These findings suggest a state of marginal stability for the large island diatom communities after the two-week colonization process, a system in presumed transition toward a future state of dynamic equilibrium as predicted by theory of island biogeography [43].

Results from analysis of Kullback-Leibler divergence show a substantial percentage difference (PD =  62.3%) associated with the process of diatom colonization and island size (small islands KL_D_: =  0.0125, large islands KL_D_ =  0.0238, Hartley units; Table 3). These findings are consistent with the observations of increased first digit analytical volatility for the larger island communities. Sample size of diatom cell counts and potential for species interactions was 70.9% greater on larger islands compared to smaller islands (Table 3), a reflection of the larger surface area available for colonization. The higher divergence of KL_D_ relative entropy associated with larger island size suggests an increase in volatility of energetic interactions among diatom species related to space competition, and/or a reduction in numbers of rare species, biotic mechanisms discussed by MacArthur and Wilson (p.56 [43]) in their review of the Patrick data. Theory of island biogeography [43] predicts diatom communities colonizing islands of varying sizes will attain steady state thresholds of species richness at different rates. The results from this case study show a similar community level functional response for counts of cells per diatom species (species evenness).

### Case Report #3. Decline of persistent amphibian populations

Published databases suitable for a Benford’s law first digit analysis, which include measurements taken before and after a loss of biodiversity, and additionally meet constraints related to the assessment of data (see Methods section), are not common in the ecological literature. One example known to this author is from Highton [44] based on visual encounters of woodland salamanders (genus *Plethodon*) spanning five decades. From 1957–1999, Highton and colleagues recorded observations of individual salamanders representing 38 species of the genus *Plethodon* from 127 sample locations in forested habitats of eastern United States. The mean number of salamanders observed per collector per site visit is given in Table 8–1 of Highton [44]. Pre-1990 surveys revealed long-term persistence for 205 *Plethodon* populations (1957 to 1988). In contrast, 1990 to 1999 surveys revealed an alarming decline in salamander numbers for many previously stable populations. While possible causes for salamander declines were not investigated, Highton reported new logging at about 12% of sample locations. Fungal infections, warming of forest temperatures from climate change, and changes in soil pH from acid rain are other suspected stressors affecting long-term survival and reproduction for woodland adapted salamander species.

Apart from the known habitat alteration associated with logging after 1988, the data meet other assessment preconditions to test correspondence with BL expectations (see Methods section). At each of the 127 sample locations, Highton and colleagues conducted daytime visual surveys of approximately one hour in sample areas about two hectares to allow for calculation of the mean number of salamander encounters per collector per site visit. Sample locations were initially selected based on the presence of abundant populations of one or more species for research on life history. The dataset spans two and three orders of magnitude, respectively. To allow for statistical comparison of similar pre- and post-1990 sampling effort, the number of site visits were normalized to 1–6 visits per collector, which was the range reported for post-1990 surveys. This normalization process resulted in first digit sample sizes of n =  96 (pre-1990) and n =  173 (post-1990) for analytical testing.

**Fig 4:**
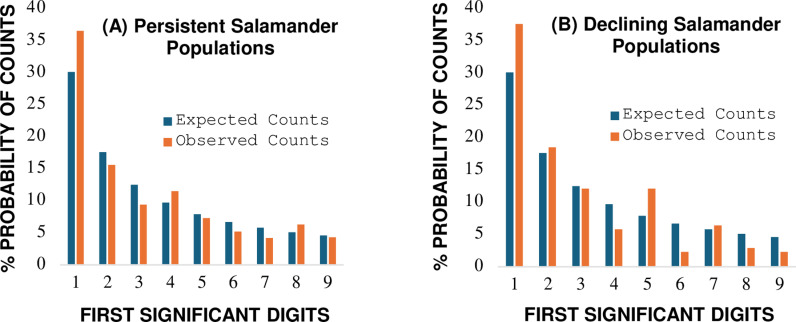
Comparison between observed first digit counts and Benford’s law expected counts for encounters of *Plethodon* salamanders from forested habitats with (A) pre-1990 persistent populations and (B) post-1990 declining populations. **Graphs of data from Table 4**.

The weight of evidence from analytical tests for pre-1990 salamander encounters (Table 4, Fig 4) support an interpretation the occurrences of 38 *Plethodon* species are well approximated by the Benford probability distribution. No signals of impending state transition away from BL reference expectations are indicated (Table 4). The results align with three decades of observations by Highton of “*consistent patterns of salamander surface activity from year to year*.” In contrast with pre-1990 data, the post-1990 results support an interpretation of statistical and descriptive non-conformity with the Benford distribution (Morrow Euclidean distance d * _n_ ≥  1.34, Kossovsky SSD ≥  100, and two Pearson residuals ≥  1.96; Table 4). The non-conformity with BL expectations aligns with observations of Highton of widespread declines in many local populations of *Plethodon* species. Between 1990 and 1999, salamander species were extirpated from 32 of 205 (15.6%) previously stable populations (Table 8–1 of Highton), with a significant reduction in mean number of salamander encounters reported (pre-1990 mean observations/site visit =  8.77, post-1990 mean =  3.65; Sign Test, χ2 =  117, *p* <  0.001).

**Table 4:** Observed first digit counts of *Plethodon* salamander encounters *vs* Benford’s law expected counts for persistent (pre-1990) and declining (post-1990) populations from eastern United States forests. (Highton [44], his Table 8–1).

First Digit	1	2	3	4	5	6	7	8	9	
Persistent Salamander populations (Pre-1990 counts)										
OBS Count	35	15	9	11	7	5	4	6	4	
EXP Count	28.9	16.9	12.0	9.3	7.6	6.4	5.6	4.9	4.4	
Pearson Residual	+1.13	−0.46	−0.86	+0.56	+0.22	−0.56	−0.66	+0.49	−0.19	
% OBS Probability	36.5	15.6	9.4	11.5	7.3	5.2	4.2	6.3	4.2	
% EXP Probability	30.1	17.6	12.5	9.7	7.9	6.7	5.8	5.1	4.6	
FSD sample size n = 96. Max Pearson residuals = 1.13 Euclidean distance d * _n_ = 0.783 (p > 0.10); Cohen-W = 0.195, 95% CI [0.193, 0.197]; Kossovsky SSD = 63.9, 95% CI [62.8, 65.0]; Kullback-Leibler divergence (0.0083 Hartley units).										
First Digit		1	2	3	4	5	6	7	8	9
**Declining Salamander populations (Post-1990 counts)**										
OBS Count		65	32	21	10	21	4	11	5	4
EXP Count		52.1	30.5	21.6	16.8	13.7	11.6	10.0	8.8	7.9
Pearson Residual		+1.79	+0.28	−0.13	−1.65	**+1.97**	**−2.23**	+0.31	−1.29	−1.39
% OBS Probability		37.6	18.5	12.1	5.8	12.1	2.3	6.4	2.9	2.3
% EXP Probability		30.1	17.6	12.5	9.7	7.9	6.7	5.8	5.1	4.6
FSD sample size n = 173; Max Pearson residuals (**1.97, 2.23**); Euclidean distance d * _n_ = **1.437** (p > 0.05); Cohen-W = 0.328, 95% CI [0.323, 0.332]; Kossovsky SSD = **119.4,** 95% CI [105.7, 133.1]; Kullback-Leibler divergence (0.0260 Hartley units). Bold values indicate non-conformity with the Benford distribution.										

Results from analysis of Kullback-Leibler divergence show an order of magnitude percentage difference (PD =  103.2%) in loss of ecological information in forested habitats associated with declining salamander abundances (pre-1990, KL_D_ =  0.0083; post 1990, KL_D_ =  0.0260, Hartley units; Table 4). Davic and Welsh [68] reviewed the roles of salamanders in forests and discuss multiple ways loss of biotic information associated with reduced numbers of *Plethodon* species can affect ecosystem processes. Key ecological functions impacted include reduced predatory regulation of resilience-resistance feedback loops associated with invertebrate prey species diversity, and reduction in structural information stored as salamander biomass, a source of high-quality and slowly available energy and nutrients for tertiary consumers over successional time.

Follow-up surveys for *Plethodon* species within the geographic range sampled by Highton [44] by Caruso and Lips [69] and Caruso et al. [70] confirm the continuation of salamander declines, in particular at sample locations dominated by larger-bodied species. The reported decline of larger sized salamanders from populations is corroborated by the BL analysis as represented by the under representation of larger first digit probabilities for post-1990 samples (Table 4, digits 6, 8, 9: pre-1990 median probability =  5.2, post-1990 median =  2.4; Mood’s Median Test, *p* =  0.014). Suggestions for future research include a BL first digit analysis for measurement variables associated with allocation of energy to growth for populations where large-bodied *Plethodon* species are under greatest selective pressure for extirpation, combined with abiotic stressor variables related to landscape fragmentation [71, 72].

### Case Report #4. Fish communities with persistent biotic integrity

For the past six decades, the State of Ohio Environmental Protection Agency (Ohio EPA) has conducted surveys of fish communities from streams and rivers to determine attainment of Federal Clean Water Act goals. Fish are sampled using standardized electrofishing protocols [45]. The overall integrity and health of fish communities is evaluated using an Index of Biotic Integrity (IBI) [73], and the agency developed Modified Index of Well-Being (MIwb). These indices are combined with information for benthic macroinvertebrate communities to assign Clean Water Act designated uses for protection of aquatic life. Stream segments designated Exceptional Warmwater Habitat (EWH) have a unique community of fish species with persistent biotic integrity associated with watersheds that experience minimal ecoregion background levels of anthropogenic disturbance [74].

Five stream segments designated EWH from various ecoregions of Ohio known to this author were randomly selected to investigate correspondence of fish species biomass with the Benford probability distribution. Details of sample locations are as follows (sample date, river mile, length of stream sampled): Grand River (2004, 8.5, 0.42 km); Chagrin River (2003, 36.4, 0.2 km); Big Darby Creek (2014, 30.2, 0.5 km); Scioto River (2011, 9.1, 0.5 km); Mohican River (2007, 6.5, 0.5 km). Fish species relative weight measurements meet data assessment constraints required to test correspondence with BL expectations (see Methods section). The location of sample zones was selected based on watershed monitoring objectives and ranged from 0.2 to 0.5 km. Fish were randomly sampled from all available habitats (riffles, runs, pools). The five stream locations yielded 67 distinct fish species along with 3 hybrids, with numerous species collected from multiple streams. The set of data spans four orders of magnitude, with total sample size sufficient for BL analysis using Morrow Euclidean distance (n ≥  80) when data from the five randomly selected EWH distributions are combined. Because the analyzed data are scattered in multiple online technical support documents, relative weight measures for the 141 fish species encounters are given in Table 5 and sorted by first digit categories. As a point of reference, the 80.82 value in Table 5 represents the relative weight (kg/km) of 156 Golden Redhorse (*Moxostoma erythrurum*) collected from the Big Darby Creek, which is ~ 27.5% of the total fish community biomass at that stream location.

**Table 5:** Relative weight (kg/km) of 141 fish species encounters from five streams in Ohio designated Exceptional Warmwater Habitat (EWH) for protection of aquatic life. Individual species measurements sorted by first digit categories. Taylor’s power law relating first digit mean and variance: Y =  3.36 X ^2.07^, r =  0.97, n =  9, *p* =  0.001 (www.statology.org/power-regression-calculator/).

First Digits		1	2	3	4	5	6	7	8	9
		0.01								
		0.01								
		0.01								
		0.01								
		0.01								
		0.01								
		0.01								
		0.01								
		0.01								
		0.01								
		0.01								
		0.01								
		0.10								
		0.11								
		0.11								
		0.11								
		0.12								
		0.14								
		0.14								
		0.14								
		0.15	0.02							
		0.16	0.02							
		0.16	0.02							
		0.16	0.02							
		0.17	0.02							
		0.17	0.02							
		0.18	0.02							
		0.19	0.02							
		1.06	0.02							
		1.08	0.02	0.03						
		1.10	0.20	0.03						
		1.20	0.20	0.03						
		1.20	0.21	0.03						
		1.52	0.29	0.03	0.04					
		1.68	2.00	0.03	0.04					
		1.71	2.00	0.03	0.04					
		1.98	2.07	0.32	0.04		0.06			
		10.52	2.23	0.34	0.04	0.05	0.06			
		11.03	2.25	3.20	0.04	0.05	0.60			
		12.00	2.41	3.20	0.40	0.05	0.63			
		14.11	2.49	3.60	0.46	0.56	0.64			
		14.72	2.80	3.68	0.48	0.56	0.64			0.09
		15.80	2.80	3.70	4.00	5.10	0.69			0.09
		16.94	2.85	32.99	4.01	5.20	6.30	0.07	0.08	0.90
		18.22	21.01	33.60	4.80	5.51	6.70	0.73	0.08	0.99
		19.50	21.30	34.30	44.90	5.67	62.25	0.76	0.87	9.00
		114.5	21.35	38.03	48.17	53.85	67.50	70.18	80.82	9.31
**Digit** **Mean**	**X**	**5.58**	**3.28**	**8.73**	**7.68**	**7.66**	**13.28**	**17.93**	**20.46**	**3.40**
**Digit** **Variance**	**Y**	**298**	**43**	**208**	**274**	**270**	**658**	**1213**	**1619**	**20**

**Fig 5:**
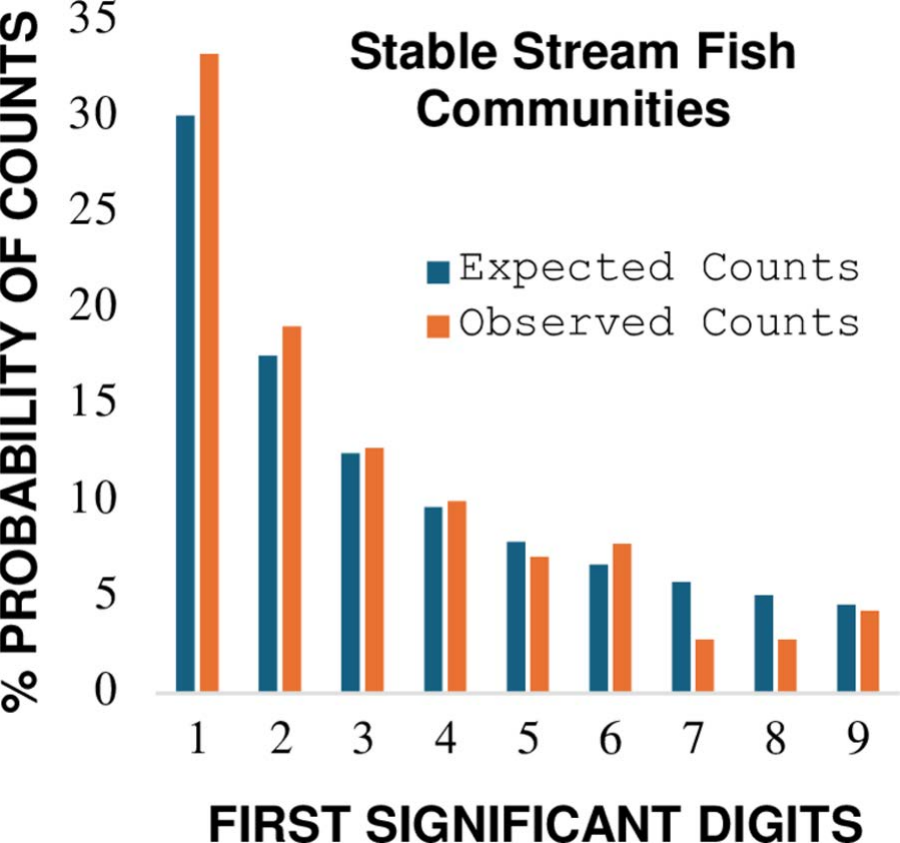
Comparison between observed first digit biomass counts and Benford’s law expected counts for species of fish collected from five geographically isolated streams with persistent and exceptional biotic integrity. **Graph of data from Table 6**.

Analytical test results (Table 6, Fig 5) support an interpretation the combined species biomass for geographically isolated EWH fish communities is well approximated by the Benford probability distribution. No signals of impending state transition away from BL reference expectations are indicated. The findings are consistent with long-term observations by Ohio EPA that EWH streams maintain persistent communities of fish over time where watersheds experience minimal background levels of human disturbance [74]. The divergence of Kullback-Leibler relative entropy of combined fish communities (0.0083 Hartley units, Table 6) is identical to the KL_D_ value recorded for 205 stable populations of *Plethodon* salamanders from forested habitats (Table 4). This observation highlights the scale invariant robustness of applying a measure of Kullback-Leibler divergence to detect spatial similarity (dissimilarity) of ecological information when applied to a BL first digit analysis.

**Table 6:** Observed first digit biomass counts *vs* Benford’ law expected counts for 141 fish species encounters from five streams in Ohio designated Exceptional Warmwater Habitat (EWH). Summary of Table 5 data.

First Digit	1	2	3	4	5	6	7	8	9
OBS Count	47	27	18	14	10	11	4	4	6
EXP Count	42.4	24.8	17.6	13.7	11.2	9.4	8.2	7.2	6.5
Pearson Residual	+0.70	+0.44	+0.09	+0.09	-0.35	+0.51	-1.46	-1.20	-0.18
% OBS Probability	33.3	19.1	12.8	10.0	7.1	7.8	2.8	2.8	4.3
% EXP Probability	30.1	17.6	12.5	9.7	7.9	6.7	5.8	5.1	4.6

FSD sample size n =  141. Max Pearson residual =  1.46; Euclidean distance d * n =  0.638 (p >  0.10); Cohen-W =  0.182, 95% CI [0.177, 0.187]; Kossovsky SSD =  28.9, 95% CI [26.4, 31.4]; Kullback-Leibler divergence (0.0083 Hartley units).

Taylor’s power law (V =  a M ^b^), where V =  variance, M =  mean, a =  constant, and b =  correlation slope, is a long studied empirical observation of‘ population mean-variance scaling related to measurements taken on organisms [75]. The mean (X) and variance (Y) of biomass measurements for the fish species from multiple persistent communities, partitioned within BL first digit categories (Table 5), reveal a strong correlation with Taylor’s power law (Y =  3.36 X ^2.07^, r =  0.97, *p* <  0.001, n =  9). The exponent (b) in the Taylor’s law equation offers insights into the spatial distribution of species, with an exponent near 2.0 associated with a more aggregated pattern of fish species behavior [75]. Both Taylor’s power law and Benford’s first digit law demonstrate scale invariance and relate to the orders of magnitude in measurement data. Future research is warranted to explore practical ecological implications of this mathematical association across various levels of organization and scale.

### Case report comparisons

**Fig 6:**
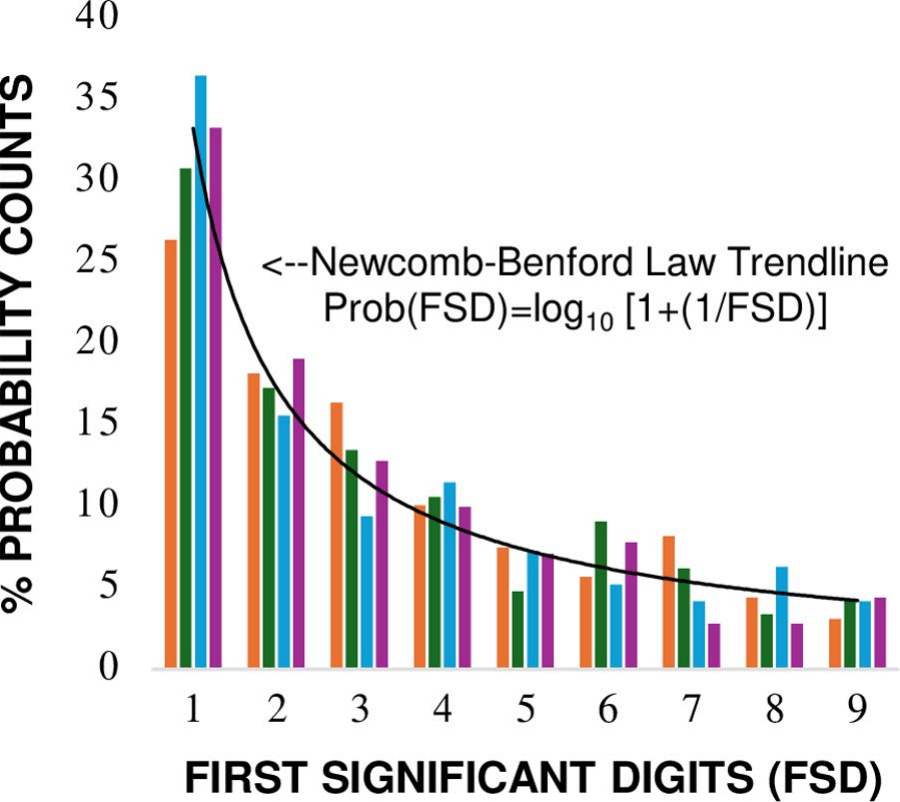
Canonical representation of the Benford probability distribution for naturally occurring multi-scale ecological systems with minimal anthropogenic disturbance in states of balanced dynamic equilibrium (case comparisons left to right: 1A, 1B, 3A, 4; Table 7).

Analytical results for seven case report comparisons are summarized in Table 7. Full statistical and descriptive conformity with the Benford probability distribution was identified for naturally occurring ecological systems in reported states of balanced dynamic equilibrium (case comparisons: 1A, 1B, 3A, 4; Table 7): Morrow Euclidean distance (0.664 < d * _N_ <  0.783), Cohen-W effect size (0.171 <  W <  0.195), Max Pearson residuals (1.13 < Max-PR <  1.59), and Kossovsky sum of square deviations (21.2 < SSD <  63.9). The ranges of analytical conformity to the Benford distribution were associated with a narrow range of informational divergence as measured by Kullback-Leibler relative entropy (0.0068 < KL_D_ <  0.0083 Hartley units, Table 7). The canonical representation of the Benford probability distribution for these stable multi-scale ecological systems with minimal anthropogenic disturbance is shown in Fig 6.

**Table 7:** Summary of analytical test results for case reports.

Case Report Comparisons	n	OM	Max PR	d * _n_	Cohen-W [95% CI]	SSD [95% CI]	KL_D_
1A: Below ground functional group biomass: early succession	159	6	1.38	0.782	0.184 [0.181,0.186]	38.4 [37.9,38.9]	0.0071
1B: Below ground functional group biomass: late succession	208	6	1.59	0.664	0.171 [0.168,0.175]	21.2 [18.7,23.7]	0.0068
2A: Experimental diatom colonization, small islands	105	6	1.87	0.759	0.250 [0.242,0.257]	54.9 [48.1,61.7]	0.0125
2B: Experimental diatom colonization, large islands	148	6	**1.96** **1.98** **	1.147	**0.316** [0.308,0.323] *	**88. 9** [79.7,98.2] *	0.0238
3A: Pre-1990 persistent salamander populations	96	2	1.13	0.783	0.195 [0.193,0.197]	63.9 [62.8,65.0]	0.0083
3B: Post-1990 declining salamander populations	173	3	**1.97** **2.23** **	**1.437** **	**0.328** [0.323,0.332] *	**119.4** [105.7,133.1] **	0.0260
4: Fish communities with exceptional biotic integrity	141	4	1.46	0.638	0.182 [0.177,0.187]	28.9 [26.4,31.4]	0.0083

FSD sample size =  n, OM =  orders of magnitude, d * _n_ =  Morrow multidimensional Euclidean distance, Max PR =  Maximum Pearson residuals, Cohen W =  Cohen-W effect size, SSD =  Kossovsky sum of square deviations. KL_D_ =  (obs||exp) Kullback-Leibler divergence (Hartley units).

* Signal of state transition either toward or away from BL steady state threshold, ** Non-conformity with Benford distribution.

A Kendall’s *tau* rank correlation analysis (GIGAcalculator.com) shows significant correlation between analytical results obtained from descriptive tests with those from multidimensional Euclidean distance (Kossovsky SSD: *tau* =  0.809, Z-score =  2.61, *p* =  0.004; Cohen-W: *tau* =  0.714, Z-score =  2.31, *p* =  0.011; Table 7 data). These findings underscore the importance of using a variety of statistical and descriptive test results to detect state transitions either toward or away from steady state thresholds approximated by the Benford probability distribution.

According to Hill’s theorem [52], the probability that empirical data will closely converge towards the Benford probability distribution is enhanced by combining sample data from multiple unbiased random distributions. This aligns with the asymptotic characteristic of the Benford distribution [59]. To empirically investigate this prediction of the Hill theorem, the combined first digit frequencies for the four naturally occurring steady state systems (case comparisons: 1A, 1B, 3A, 4; Table 7) were analytically tested against BL expectations. Test results provide empirical ecological data in support of the Hill theorem (Table 8), best evidenced from the significantly lower confidence interval ranges for Cohen-W effect size and Kossovsky SSD as compared to values for individual case report comparisons (see Table 7).

**Table 8:** Observed first digit counts *vs* Benford’s law expected counts for multi-scale ecological systems in reported steady state dynamic equilibrium. Data compiled from case comparisons (1A, 1B, 3A, 4; Table 7).

First Digit	1	2	3	4	5	6	7	8	9
OBS Count	188	107	81	63	39	44	34	24	24
EXP Count	181.8	106.4	75.5	58.5	47.8	40.4	35.0	30.9	27.6
Pearson Residual	+0.46	+0.06	+0.64	+0.58	-1.28	+0.56	-0.17	-1.24	-0.69
% OBS Probability	31.1	17.7	13.4	10.4	6.5	7.3	5.6	4.0	4.0
% EXP Probability	30.1	17.6	12.5	9.7	7.9	6.7	5.8	5.1	4.6

FSD sample size n =  604; Max Pearson residual =  1.28; Euclidean distance d * _n_ =  0.632 (p >  0.01); Cohen-W =  0.091, 95% CI [0.088,0.093]; Kossovsky SSD =  6.62, 95% CI [6.56, 6.68]; Kullback-Leibler divergence (KL_D_ =  0.0019).

## Discussion

This study presents empirical evidence from reviewed case reports demonstrating a statistical relationship between the pattern of first significant digit probabilities described by the Newcomb-Benford number law [8–10] and naturally occurring ecological systems in balanced states of dynamic equilibrium (Table 7, Fig 6). MacArthur [76] observed that the collection of empirical data in ecology is often an exercise of identifying recurring mathematical patterns in nature. Margalef (p. 12, [77]) presented an interpretation of ecological stability based on the frequency of numeric fluctuations for ratio of production/biomass (P/B), such that a lower frequency of (P/B) fluctuation equates to higher system stability. The case study results presented here lend support to this cybernetic perspective of Margalef. Non-additive measurement variables associated with multi-scale ecological systems in steady state dynamic equilibrium show consistently lower fluctuation in the frequencies of first digit probabilities described by the Benford probability distribution (Table 7, Fig 6). The empirical phenomenon is scale-invariant [10,28] and documented at multiple levels of ecological organization (Figs 2–5). The correspondence aligns with measurements taken on multiple physical and social systems [11–25], mathematical constants of nature [78], thermodynamic theory [27, 28], and anecdotal ecological observations [25,34–38]. Analytical test results from case reports (Table 7) highlight the importance of utilizing both statistical and descriptive approaches to enhance the likelihood of identifying a signal of state transition either toward or away from the reference approximation of system consistency associated with the Benford distribution. It is important to note, however, that many naturally occurring ecological datasets may not follow the BL leading digit pattern. Analytical conformity is subject to multiple constraints related to the data assessment process, as outlined in the Methods section.

A novel observation from case reports is that a Benford’s law first digit analysis of empirical data can be applied to detect temporal and spatial divergence of ecological information through the measurement of Kullback-Leibler relative entropy [63], in both real-time and time-series domains. A narrow range of Kullback-Leibler divergence between observed first digit probabilities and those expected by the Benford distribution (lower uncertainty and higher predictability of stability) is associated with ecological systems showing minimal evidence of anthropogenic disturbance of natural habitats (case comparisons 1A, 1B, 3A, 4; KL_D_ range: 0.0068 to 0.0083 Hartley units; Table 7). Conversely, order of magnitude higher KL_D_ values (higher uncertainty and lower predictability of stability) are associated with non-conformity to the Benford distribution (comparisons 2B, 3B; KL_D_ range 0.0238 to 0.0260 Hartley units; Table 7). Huckeba et al. [65] review ecological applications of Kullback-Leibler divergence including its use as a theoretical framework for cross-scale analysis of ecological landscapes, as an early warning signal to detect tipping points for complex diseases, and for network analysis to monitor node-edge dynamics in response to system perturbations. Burgos and Santos [28] applied Kullback-Leibler relative entropy to the first digit probabilities of the Benford distribution to investigate the scale invariant properties of Markovian processes, which are common in biological systems [53]. The data presented here, derived from a BL first digit analysis of empirical data, suggest future application of Kullback-Leibler divergence to help quantify the entropy production and dissipation of energy [34] associated with multiple ecological processes (i.e., secondary production, metabolism, decomposition rates, nutrient cycling, food-web feedback interactions, etc.) that function to maintain steady state behavior.

Analytical results from a diatom colonization experiment using artificial glass slides for cell attachment (case comparison 2A, Table 7) show full statistical conformity to the Benford distribution, but also an order of magnitude increase of Kullback-Leibler divergence when compared to stable systems associated with natural habitats (case comparisons 1A, 1B, 3A, 4; Table 7). These findings suggest that statistical and informational conformity to steady state conditions may reflect distinct ecological phenomena when viewed through the lens of first significant digit probabilities. The documentation of a significant divergence of Kullback-Leibler relative entropy for a system judged to be statistically stable implies veiled informational disorder of internal system processes related to dissipation of energy and lower predictability of future behavior [34]. O’Connor et al. [64] discuss how the statistical collapse of ecosystem stability may follow a more subtle prior collapse of ecological information but were unaware of sampling techniques appropriate for hypothesis testing. The Benford’s law leading digit assessment protocol outlined here offers a robust analytical approach to investigate this bifurcated collapse phenomenon by combining statistical and descriptive analytical test results for measurement variables with information from Kullback-Leibler divergence, perhaps in conjunction with other informational statistics such as Rènyi entropy and Tsallis entropy that may be more sensitive to system perturbations expected for complex adapted systems. As presented by O’Connor et al. [64], information in this context would refer to multiple ecological processes, which “encode, filter, and process information stored in biological structure, as well as semiotic information associated with structures and their content.”

Ecological ‘systems’ typically are identified by interactions of matter and energy at the population, community, and ecosystem levels of organization [79]. The documentation of conformity to the Benford distribution for the geographically isolated distributions of fish communities (case comparison #4, Table 7), and widespread populations of woodland salamanders (comparison #3A), is consistent with the meta-distribution concept of system organization discussed by Leibold et al [80]. While typically meta-distributions of fauna are linked by matter and energy via migrations of individuals, additional linkage from shared ecological information [64] is suggested by the similarity of Kullback-Leibler relative entropy for the fish communities and salamander populations (Table 7). By applying a BL first digit analysis to meta-distribution datasets, the divergence of Kullback-Leibler information among sample distributions in states of balanced dynamic equilibrium would be expected to be minimized where constraints associated with the assessment of empirical data are maximized (see Methods section). The intriguing topic of information flow and its relation to meta-distribution ecosystem dynamics is reviewed by Little et al. [81].

In addition to ecological processes related to dynamic equilibrium, attributes of system complexity and maturity also can be identified from a BL leading digit analysis by examining pattern in the set of raw data measurements partitioned into first digit categories. For example, biomass measurements for encounters of fish species from multiple stable stream communities (case report #4, Table 5) reveal a skewed pattern resembling diversity curves for a variety of ecological measurement variables (see Odum, p.149 [79]), as well as a Type-C survivorship curve (Odum, p.174 [79]), which is identified with younger and older age classes. The same dataset shows strong correlation between mean-variance scaling as predicted by Taylor’s power law (see Table 5), a phenomenon more commonly associated with the population level of organization [75]. Moreover, the first digit *probabilities* for the dataset (Table 6) allow for computation of scale-invariant indices of system complexity, such as the 1949 Simpson Diversity Index [82], which can be tailored for application to the Benford distribution by replacing (*K*) categories of species with (*K*) categories of first digit probabilities:

$$Simpson\text{ Diversity }Index\text{ D =}\overset{9}{\underset{K=1}{\sum }}{\left(d\right)}^{2}$$ (7)

where (*d*) is the probability of first digit counts (1:9) appearing within one of the nine (*K*) first digit categories. As a reference approximation, the expected first digit probabilities of the Benford distribution yields a Simpson Diversity Index of D =  0.165 (Table 1 data). This unconventional application of the Simpson index advances a holistic approximation of system complexity derived from invariant multi-scale first digit probabilities, independent of taxonomic identity. For instance, the *observed* first digit probabilities of fish biomass measurements from Table 6 (0.333, 0.191, 0.128…) yields a Simpson Index (D =  0.187), which can be applied to monitor the time-series transition of biomass diversity at a meta-community level of organization, as well as provide a real-time estimate of percentage difference of system diversity relative to the BL reference approximation expected under maximum steady state conditions (D =  0.165). In the context of a BL leading digit assessment, the Simpson Diversity Index (Eq. 7) ranges from 1/*K* (1/9 =  ~  0.111) to 9/K (9/9 =  1.0). Simpson D values closer to the lower threshold are associated with a more uniform pattern of first digit probabilities and a higher level of system diversity for the measurement variable of interest [82].

A growing number of studies report correspondence of ecological, genetic, and evolutionary processes with the Benford’s law pattern of first digit probabilities [17,18,25,37–41]. Additional avenues for future investigation include: 1) analysis of multiple measurement variables at different organizational levels and scale; 2) comparative methodologies for random sampling and statistical analysis; 3) controlled micro- and mesocosm experiments to assess the impact of human perturbations on environmental conditions; 4) examination of large datasets generated through remote sensing [83]; 5) forecasting analysis to detect imminent state transitions in real-time and to test the realism of ecological models that emulate empirical data [37]; and 6) the use of detrital metabolism as a measurement variable, suggested by Wetzel (p.703 [84]) to be a fundamental driver of stability for both aquatic and terrestrial ecosystems. Future investigation also is warranted to study correspondence of BL expectations with MacArthur’s broken stick model of niche appointments [85], theory of maximum entropy [33,86], ascendency model of Ulanowicz [35], and effective complexity as a compressed vector of first digit frequencies [87]. Lemons [27] documented the Benford distribution emerges from an unbiased partitioning of a set of conserved physical quantities into the nine first digit categories for thermodynamically isolated systems, a finding in need of validation for open ecological systems. Frank [88] reviewed the primacy of invariances to aid understanding of probability patterns observed in nature, which, given the scale invariant characteristic of the Benford distribution [28], holds potential for future theoretical inquiry.

Consensual agreement on the measurement of ecological stability remains elusive, with the prevailing view of Landi et al. [89] that no single system measurement, such as species diversity, can aggregate all historic interpretations of the stability concept. Ives and Carpenter [90] advocate for a holistic approach: “*it will be more profitable to study stability comprehensively, including diversity as only one of the possible factors that affect ecosystem responses to environmental change.*” A Benford’s law analysis of empirical data shows utility to investigate the comprehensive stability of complex adapted systems. By analyzing the differences between observed and expected first digit probabilities for various *measurement variables* (i.e., species richness and evenness, biomass, metabolism, decomposition, etc.), researchers can detect natural self-organizing patterns and anomalies associated with ecological processes. The integration of additional system information from *statistical parameters*, such as the Simpson Diversity Index, indices of Biotic Integrity, and Kullback-Leibler divergence facilitates development of a comprehensive theoretical framework (*sensu* Ives and Carpenter) to investigate how ecological processes related to stability, complexity, and maturity simultaneously respond to natural and human-induced environmental changes.

Hastings et al. [91] review the importance of applying ‘quantitative reasoning’ and development of new mathematical approaches to address the question of how best to simplify and extract information from complex adapted systems. They identified three interacting themes for future inquiry (nonlinearity, stochastic processes, scale of time and place) all of which are fundamental to, and integrated by, the mathematical properties of the Benford probability distribution. To this list can be added characteristics of the Benford distribution related to predictability of abnormal behavior and robustness to prior assumptions regarding measurement preconditions. A simple first significant digit transformation of arithmetic data randomly collected on organisms, reassembled into a representation of logarithmic probabilities described by the Newcomb-Benford number law, provides unique opportunities to apply quantitative reasoning to advance our understanding of ecological processes that organize the complex adapted systems of nature.

## Conclusion

Analytical results obtained from constrained sets of empirical data reveal a scale invariant statistical correspondence between the Benford probability distribution and ecological systems in states of balanced dynamic equilibrium. Attributes of system complexity and maturity can be investigated by examining pattern in measurement variables partitioned into first significant digit categories of information. Results from case studies highlight novel applications of the Benford distribution to detect impending state transition of marginally stable systems, as well as temporal and spatial divergence of ecological information through the measurement of Kullback-Leibler relative entropy. Widespread documentation of the leading digit phenomenon is expected as ecologists revisit historic sets of quantitative data and formalize sampling protocols for its detection. The conversion of empirical sets of data into logarithmic probabilities of leading digits described by the Newcomb-Benford number law provides unique opportunities to advance our understanding of ecological processes related with stability, complexity, and maturity.

## Study limitations

The information presented here has a number of limitations, foremost is the small number of case comparisons presented for hypothesis testing. There is, however, a marked deficiency in the availability of published ecological data that adheres to the assessment pre-conditions essential for exploring the Newcomb-Benford law phenomenon. Only one measurement variable was analyzed for each case report, yet complex adapted systems may show stability for one variable and instability in another. Future analysis of multiple measurement variables simultaneously (biomass, species richness and evenness, metabolism, productivity, decomposition rates, nutrient cycling, trophic dynamics, etc.) will enhance our understanding of correspondence of ecological processes with the Benford probability distribution. It is important to note that not all biotic systems in steady state dynamic equilibrium that are well described by the Benford distribution will necessarily be of beneficial use to humans. Examples include lentic habitats with seasonally consistent blooms of cyanobacteria reaching limits of toxicity to wildlife, and biotic communities with dominant and persistent populations of exotic species that can alter the distribution and abundance of native species to attain alternate states of dynamic equilibrium away from conditions prior to human influence. The Benford probability distribution is an empirical observation elevated to the level of a testable hypothesis due to its capacity to detect the emergence of scale invariant logarithmic patterns within naturally occurring systems, and to signal abnormal system behaviors. Nonetheless, the underlying mechanisms linking specific first digit probabilities to ecological processes are unknown. Controlled micro-and mesocosm experiments using multiple measurement variables are suggested for future theoretical inquiry.

## References

[1] Hutchinson GE. The concept of pattern in ecology. Proc Acad Natural Sci Phil. 1953;105:1–12.

[2] Levin SA. Self-organization and the emergence of complexity in ecological systems. BioScience. 2005;55(12):1075. doi: 10.1641/0006-3568(2005)055[1075:sateoc]2.0.co;2

[3] Brown JH, Gupta VK, Li B-L, Milne BT, Restrepo C, West GB. The fractal nature of nature: power laws, ecological complexity and biodiversity. Philos Trans R Soc Lond B Biol Sci. 2002;357(1421):619–26. doi: 10.1098/rstb.2001.0993 12079523 PMC1692973

[4] Marquet PA, Quiñones RA, Abades S, Labra F, Tognelli M, Arim M, et al. Scaling and power-laws in ecological systems. J Exp Biol. 2005;208(Pt 9):1749–69. doi: 10.1242/jeb.01588 15855405

[5] Psillos S. Regularities, natural patterns, and laws of nature. Theoria. 2014;29(1):9–27. doi: 10.1387/THEORIA.8991

[6] McAllister JW. What do patterns in empirical data tell us about the structure of the world?. Synthese. 2011;182(1):73–87. doi: 10.1007/s11229-009-9613-x

[7] Dodds K. Laws, theories, and patterns in ecology. Berkeley: University of California Press;2009.

[8] Newcomb S. Note on the frequency of use of the different digits in natural numbers. Amer J Math. 1881;4(1):39–40.

[9] Benford F. The law of anomalous numbers. Proc Amer Philos Soc. 1938;78(4):551–72.

[10] Pinkham RS. On the distribution of first significant digits. Ann Math Statist. 1961;32(4):1223–30. doi: 10.1214/aoms/1177704862

[11] Diaz J, Gallart J, Ruiz M. On the ability of the Benford’s law to detect earthquakes and discriminate seismic signals. Seismol Res Lett. 2014;86(1):192–201. doi: 10.1785/0220140131

[12] Seenivasan P, Easwaran S, Sridhar S, Sinha S. Using skewness and the first-digit phenomenon to identify dynamical transitions in cardiac models. Front Physiol. 2016;6:390. doi: 10.3389/fphys.2015.00390 26793114 PMC4707587

[13] Nigrini MJ. Benford’s law: Applications for Forensic Accounting, Auditing, and Fraud Detection. Hoboken. New Jersey: Wiley Online Library; 2012.

[14] Kreuzer M, Jordan D, Antkowiak B, Drexler B, Kochs EF, Schneider G. Brain electrical activity obeys Benford’s law. Anesth Analg. 2014;118(1):183–91. doi: 10.1213/ANE.0000000000000015 24356167

[15] Li Q, Fu Z. Quantifying non-stationarity effects on organization of atmospheric turbulent eddy motion by Benford’s law. Commun Nonlinear Sci Nume Simulat. 2016;33:91–8. doi: 10.1016/j.cnsns.2015.09.006

[16] Villas-Boas S, Fu Q, Judge G. Is Benford’s law a universal behavioral theory?. Econometrics. 2015;3(4):698–708. doi: 10.3390/econometrics3040698

[17] Friar JL, Goldman T, Pérez-Mercader J. Genome sizes and the Benford distribution. PLoS One. 2012;7(5):e36624. doi: 10.1371/journal.pone.0036624 22629319 PMC3356352

[18] Docampo S, del Mar Trigo M, Aira MJ, Cabezudo B, Flores-Moya A. Benford’s law applied to aerobiological data and its potential as a quality control tool. Aerobiologia. 2009;25(4):275–83. doi: 10.1007/s10453-009-9132-8

[19] Nigrini MJ, Miller SJ. Benford’s law applied to hydrology data—results and relevance to other geophysical data. Math Geol. 2007;39(5):469–90. doi: 10.1007/s11004-007-9109-5

[20] Sen D, Sen U. Benford’s law detects quantum phase transitions similarly as earthquakes. Europhys Lett. 2011;95 50008. doi: 10.1209/0295-5075/95/50008

[21] Li Q, Fu Z, Yuan N. Beyond Benford’s law: distinguishing noise from chaos. PLoS One. 2015;10(6):e0129161. doi: 10.1371/journal.pone.0129161 26030809 PMC4452586

[22] Sambridge S, Tkalčić H, Jackson A. Benford’s law in the natural sciences. Geophys Res Lett. 2010;37(22):l22301. doi: 10.1029/2010GL044830

[23] Muchnik F-B. Benford’s law and geographical information - the example of OpenStreetMap. Int J Geogr Info Sci. 2021;35(9):1746–72. doi: 10.1080/13658816.2020.1829627

[24] Yang L, Fu Z. Out-phased decadal precipitation regime shift in China and the United States. Theor Appl Climatol. 2016;130(1–2):535–44. doi: 10.1007/s00704-016-1907-6

[25] Costas E, López-Rodas V, Toro FJ, Flores-Moya A. The number of cells in colonies of the cyanobacterium Microcystis aeruginosa satisfies Benford’s law. Aqua Bot. 2008;89(3):341–3. doi: 10.1016/j.aquabot.2008.03.011

[26] Thoms MC, Piégay H, Parsons M. What do you mean, ‘resilient geomorphic systems’?. Geomorphology. 2018;305:8–19. doi: 10.1016/j.geomorph.2017.09.003

[27] Lemons DS. Thermodynamics of Benford’s first digit law. Am J Phys. 2019;87(10):787–90. doi: 10.1119/1.5116005

[28] Burgos A, Santos A. The newcomb–Benford law: scale invariance and a simple Markov process based on it. Amer J Phys. 2021;89(9):851–61. doi: 10.1119/10.000495

[29] Kay JJ, Schneider ED. Thermodynamics and measures of ecological integrity. In: McKenzie DH, Hyatt DE, McDonald VJ, editors. Ecological Indicators. Boston: Springer;1992. 159–82. doi: 10.1007/978-1-4615-4659-7-12

[30] Chapman EJ, Childers DL, Vallino JJ. How the second law of thermodynamics has informed ecosystem ecology through its history. BioScience. 2015;66(1):27–39. doi: 10.1093/biosci/biv166

[31] Nielsen SN, Müller F, Marques JC, Bastianoni S, Jørgensen SE. Thermodynamics in ecology-an introductory review. Entropy (Basel). 2020;22(8):820. doi: 10.3390/e22080820 33286591 PMC7517404

[32] Meysman FJR, Bruers S. A thermodynamic perspective on food webs: quantifying entropy production within detrital-based ecosystems. J Theor Biol. 2007;249(1):124–39. doi: 10.1016/j.jtbi.2007.07.015 17720204

[33] Harte J, Newman EA. Maximum information entropy: a foundation for ecological theory. Trends Ecol Evol. 2014;29(7):384–9. doi: 10.1016/j.tree.2014.04.009 24863182

[34] Prigogine I, Nicolis G. Biological order, structure and instabilities. Q Rev Biophys. 1971;4(2):107–48. doi: 10.1017/s0033583500000615 4257403

[35] de Castro C, McShea DW. Applying the Prigogine view of dissipative systems to the major transitions in evolution. Paleobiology. 2022;48(4):711–28. doi: 10.1017/pab.2022.7

[36] Ulanowicz RE. Some steps toward a central theory of ecosystem dynamics. Comput Biol Chem. 2003;27(6):523–30. doi: 10.1016/s1476-9271(03)00050-1 14667780

[37] Özkundakci D, Pingram MA. Nature favours “one” as the leading digit in phytoplankton abundance data. Limnologica. 2019;78:125707. doi: 10.1016/j.limno.2019.125707

[38] Campos L, Salvo AE, Flores-Moya A. Natural taxonomic categories of angiosperms obey Benford’s law, but artificial ones do not. Syst Biodivers. 2016;14(5):431–40. doi: 10.1080/14772000.2016.1181683

[39] Pröger L, Griesberger P, Hackländer K, Brunner N, Kühleitner M. Benford’s law for telemetry data of wildlife. Stats. 2021;4(4):943–9. doi: 10.3390/stats4040055

[40] Szabo JK, Forti LR, Callaghan CT. Large biodiversity datasets conform to Benford’s law: Implications for assessing sampling heterogeneity. Biol Conserv. 2023;280:109982. doi: 10.1016/j.biocon.2023.109982

[41] Shim T, Kim Z, Jung J. A Benford’s law-based framework to determine the threshold of occurrence sites for species distribution modelling from ecological monitoring databases. Sci Rep. 2023;13(1):16777. doi: 10.1038/s41598-023-44010-z 37798344 PMC10556063

[42] Neutel A-M, Heesterbeek JAP, van de Koppel J, Hoenderboom G, Vos A, Kaldeway C, et al. Reconciling complexity with stability in naturally assembling food webs. Nature. 2007;449(7162):599–602. doi: 10.1038/nature06154 17914396

[43] MacArthur RH, Wilson EO. The theory of island biogeography. Princeton. New Jersey: Princeton University Press, Monographs in Population Biology; 1967.

[44] Highton R. Declines of eastern North American woodland salamanders (Plethodon). in: Lannoo M, editor. Amphibian Declines: The Conservation Status of United States Species. . Los Angeles, California: University of California Press;2005:34–46.

[45] Ohio Environmental Protection Agency. Biological criteria for the protection of aquatic life, Vol III: Standardized biological field sampling and laboratory methods for assessing fish and macroinvertebrate communities. Ecological Assessment Section, Division of Surface Water. Columbus, Ohio;2015 revision. Available from: http://www.epa.ohio.gov/portals/35/cocuments/BioCrit15_Vol3.pdf.

[46] Hill TP. Base-Invariance Implies Benford’s Law. Proc Amer Math Soc. 1995;123(3):887. doi: 10.2307/2160815

[47] Durtschi C, Hillison W, Pacini C. The effective use of Benford’s law to assist in detecting fraud in accounting data. J Forens Acct. 2004;5(1):17–34.

[48] Berger A, Hill TP. The mathematics of Benford’s law: a primer. Stat Methods Appl. 2020;30(3):779–95. doi: 10.1007/s10260-020-00532-8

[49] Miller SJ. Benford’s law: theory and applications. Princeton, New Jersey: Princeton University Press;2015.

[50] Fang G, Chen Q. Several common probability distributions obey Benford’s law. Physica A: Statis Mech Applica. 2020;540:123129. doi: 10.1016/j.physa.2019.123129

[51] Berger A, Hill TP, Rogers E. Benford Online Bibliography;2024. [Internet] Available from: https://www.benfordonline.net/.

[52] Hill TP. A statistical derivation of the significant-digit law. Statist Sci. 1995;10(4). doi: 10.1214/ss/1177009869

[53] Ashby WR. An Introduction to Cybernetics. London, United Kingdom: Methuen and Company, LTD;1979.

[54] Zwick M. Information, constraint and meaning. proceedings of society for general system research. International Conference, NYC. Smith AW, editor. Intersystems Publication;1984:93–9.

[55] Fewster RM. A Simple Explanation of Benford’s Law. Amer Statis. 2009;63(1):26–32. doi: 10.1198/tast.2009.0005

[56] Boyle J. An application of Fourier series to the most significant digit problem. Amer Math Month. 1994;101(9):879–86.

[57] Benjamini Y, De Veaux R, Efron B, Evans S, Glickman M, Graubard B. The ASA President’s task force statement on statistical significance and replicability. Ann Appl Statis. 2021;15(3):1084–5. doi: 10.124/21-AOAS1501

[58] Tam Cho WK, Gaines BJ. Breaking the (Benford) law: statistical fraud detection in campaign finance. Amer Statis. 2007;61(3):218–23. doi: 10.1198/000313007x223496

[59] Morrow J. Benford’s law, family of distributions, and a test basis. Center for Economic Performance, London School of Economics, UK; 2014: discussion paper 1291. [Internet] Available from: https://www.johnmorrow.info/projects/benford/benfordMain.pdf

[60] Kossovsky A. On the mistaken use of the chi-square test in Benford’s law. Statistics. 2021;4(2):419–53. doi: 10.3390/stats4020027

[61] Cohen J. Statistical Power Analysis for the Behavioral Sciences. 2nd ed. Hillsdale, New Jersey: Lawrence Erlbaum Associates;1988.

[62] Sharpe D. Your chi-square test is statistically significant: now what?. Pract Assess Res Eval. 2015;20:1–10.

[63] Kullback S, Leibler RA. On information and sufficiency. Ann Math Statist. 1951;22(1):79–86. doi: 10.1214/aoms/1177729694

[64] O’Connor MI, Pennell MW, Altermatt F, Matthews B, Melián CJ, Gonzalez A. Principles of ecology revisited: integrating information and ecological theories for a more Unified Science. Front Ecol Evol. 2019;7. doi: 10.3389/fevo.2019.00219

[65] Huckeba G, Andresen B, Roach T. Multi-scale spatial ecology analyses: a Kullback information approach. Land Ecol. 2023;38(3):645–57. doi: 10.1007/s10980-022-01514-9

[66] van Altena C, Hemerik L, de Ruiter P. Food web stability and weighted connectance: the complexity-stability debate revisited. Theor Ecol. 2016;9 49–58. doi: 10.1007/s12080-015-0291-7

[67] Patrick R. The formation and maintenance of benthic diatom communities. Proc Amer Philos Soc. 1976;120(6):475–84.

[68] Davic RD, Welsh HH Jr. On the ecological roles of salamanders. Annu Rev Ecol Evol Syst. 2004;35(1):405–34. doi: 10.1146/annurev.ecolsys.35.112202.130116

[69] Caruso N, Lips K. Truly enigmatic declines in terrestrial salamander populations in Great Smoky Mountains National Park. Diversity Distrib. 2013;19(1–2):38–48. doi: 10.1111/j.1472-4642.2012.00938.x

[70] Caruso NM, Sears MW, Adams DC, Lips KR. Widespread rapid reductions in body size of adult salamanders in response to climate change. Glob Chang Biol. 2014;20(6):1751–9. doi: 10.1111/gcb.12550 24664864

[71] Halley JM, Pimm SL. The rate of species extinction in declining or fragmented ecological communities. PLoS One. 2023;18(7):e0285945. doi: 10.1371/journal.pone.0285945 37437089 PMC10337920

[72] Ramírez-Carrillo E, López-Corona O, Toledo-Roy JC, Lovett JC, de León-González F, Osorio-Olvera L, et al. Assessing sustainability in North America’s ecosystems using criticality and information theory. PLoS One. 2018;13(7):e0200382. doi: 10.1371/journal.pone.0200382 30011317 PMC6047788

[73] Karr JR, Dudley DR. Ecological perspective on water quality goals. Environmental Management. 1981;5(1):55–68. doi: 10.1007/bf01866609

[74] Ohio Environmental Protection Agency. Summary of findings from the 2020-2021 aquatic life and water quality surveys of Ohio’s large rivers. Division of Surface Water;2004. Ohio EPA Technical Report: AMS/2020-LRGRV-2. Available from: https://dam.assets.ohio.gov/image/upload/epa.ohio.gov/Portals/35/tmdl/LargeRiverSurvey-DataSummary-2023.pdf.

[75] Kendal WS. Taylor’s ecological power law as a consequence of scale invariant exponential dispersion models. Ecol Complex. 2004;1(3):193–209. doi: 10.1016/j.ecocom.2004.05.001

[76] MacArthur RH. Geographical Ecology: Patterns in the Distribution of Species. Princeton. New Jersey: Princeton University Press;1984.

[77] Margalef R. Perspectives in Ecological Theory. Chicago. Illinois: The University of Chicago Press;1968.

[78] Burke J, Kincanon E. Benford’s law and physical constants: The distribution of initial digits. Amer J Phy. 1991;59(10):952–952. doi: 10.1119/1.16838

[79] Odum EP. Fundamentals of Ecology. 3rd ed. Philadelphia, Pennsylvania: W.B. Saunders Co; 1971.

[80] Leibold MA, Holyoak M, Mouquet N, Amarasekare P, Chase JM, Hoopes MF. The metacommunity concept: a framework for multi-scale community ecology. Ecol Lett. 2004;7(7):601–13. doi: 10.1111/j.1461-0248.2004.00608.x

[81] Little CJ, Rizzuto M, Luhring TM, Monk JD, Nowicki R, Paseka RE, et al. Filling the information gap in meta-ecosystem ecology. [Internet]. ResearchGate preprint;2020. doi: 10.32942/osf.10/hc83u

[82] Simpson E. Measurement of diversity. Nature. 1949;163:688.

[83] Farley S, Dawson A, Goring S, Williams J. Situating ecology as a big-data science: Current advances, challenges, and solutions. Bioscience. 2018;68(8):563–76. doi: 10.1093/biosci/biy068

[84] Wetzel RG. Limnology. 2nd Ed. Philadelphia, Pennsylvania: Saunders College Publishing;1983.

[85] Tokeshi M. Species Abundance Patterns and Community Structure. Advances in Ecological Research. 1993:111–86. doi: 10.1016/s0065-2504(08)60042-2

[86] Harte J. Maximum entropy and ecology: a theory of abundance, distribution, and energetics. Oxford, United Kingdom: Oxford University Press;2011.

[87] Gell-Mann M, Lloyd S. Information measures, effective complexity, and total information. Complexity. 1996;2(1):44–52. doi: 10.1002/(sici)1099-0526(199609/10)2:1

[88] Frank SA. The invariances of power law size distributions. F1000Res. 2016;5:2074. doi: 10.12688/f1000research.9452.2 27928497 PMC5115223

[89] Landi P, Minoarivelo HO, Brännström Å, Hui C, Dieckmann U. Complexity and stability of ecological networks: a review of the theory. Pop Ecol. 2018;60(4):319–45. doi: 10.1007/s10144-018-0628-3

[90] Ives AR, Carpenter SR. Stability and diversity of ecosystems. Science. 2007;317(5834):58–62. doi: 10.1126/science.1133258 17615333

[91] Hastings A, Arzberger P, Bolker B, Collins S, Ives A, Johnson N, et al. Quantitative bioscience for the 21st century. Bioscience. 2005;55(6):511–7. doi: 10.1641/0006-3568(2005)055[0511:QBFTSC]2.0.CO;2

[92] Davic RD. Correspondence of Newcomb-Benford number law with ecological processes. bioRxiv. 2022. doi: 10.1101/2022.06.27.497806 PMC11952760 40153682

